# Comparison of intraspecific, interspecific and intergeneric chloroplast diversity in Cycads

**DOI:** 10.1038/srep31473

**Published:** 2016-08-25

**Authors:** Guo-Feng Jiang, Damien Daniel Hinsinger, Joeri Sergej Strijk

**Affiliations:** 1 Plant Ecophysiology and Evolution Group, State Key Laboratory for Conservation and Utilization of Subtropical Agro-Bioresources and College of Forestry, Guangxi University, Nanning, Guangxi 530004, China

## Abstract

Cycads are among the most threatened plant species. Increasing the availability of genomic information by adding whole chloroplast data is a fundamental step in supporting phylogenetic studies and conservation efforts. Here, we assemble a dataset encompassing three taxonomic levels in cycads, including ten genera, three species in the genus *Cycas* and two individuals of *C. debaoensis*. Repeated sequences, SSRs and variations of the chloroplast were analyzed at the intraspecific, interspecific and intergeneric scale, and using our sequence data, we reconstruct a phylogenomic tree for cycads. The chloroplast was 162,094 bp in length, with 133 genes annotated, including 87 protein-coding, 37 tRNA and 8 rRNA genes. We found 7 repeated sequences and 39 SSRs. Seven loci showed promising levels of variations for application in DNA-barcoding. The chloroplast phylogeny confirmed the division of Cycadales in two suborders, each of them being monophyletic, revealing a contradiction with the current family circumscription and its evolution. Finally, 10 intraspecific SNPs were found. Our results showed that despite the extremely restricted distribution range of *C. debaoensis*, using complete chloroplast data is useful not only in intraspecific studies, but also to improve our understanding of cycad evolution and in defining conservation strategies for this emblematic group.

Cycads are iconic relict species, regarded as “living fossils” because of their recognizable intermediate morphological traits between angiosperms and gymnosperms^1^. Cycads dominated the Mesozoic but their origin can be dated to the late Paleozoic (~265–290 Ma)^2^,^3^. However, molecular dating studies indicate that living cycad species could be not much older than ~12 Ma, rejecting both the hypothesized role of dinosaurs in generating extant diversity and the use of “living fossils” to describe current cycad species^4^. Cycads are distributed in tropical and subtropical regions of Africa, Asia, Oceania and America^5^. Ten genera and 344 species are currently accepted^6^, with *Cycas* containing roughly 40% of the species in the Near Threatened and Vulnerable categories in the IUCN red list^7^.

The genus *Cycas* L., in the monotypic family Cycadaceae, is the oldest genus of cycads, holding about 113 species^5^. More than 20 species are found in China^5^,^8^, with most of them endemic. *Cycas debaoensis* Y. C. Zhong & C. J. Chen, a critically endangered cycad species endemic to southwest China^9^, only occurs in 11 small populations near the border of Guangxi province and Yunnan province^10^. Previous studies have assessed genetic diversity in *C. debaoensis* using inter simple sequence repeat (ISSR) markers or nuclear microsatellites, showing limited gene flow among populations and low within-population diversity^10^,^11^,^12^.

Chloroplasts (cps) are present in photosynthetically active green tissues and generally develop from proplastids in meristems or etioplasts after illumination of dark-grown tissues, and display a conserved structure of two inverted repeats (IR) separated by small (SSC) and large (LSC) single-copy regions^13^. Due to their natural abundance in plant cells (≈3–5% of the cell DNA content comparing to nuclear DNA)^14^, cp sequences are a versatile tool for plant identification (DNA-barcoding) and evolutionary studies. They have been used at small and large temporal scales in plants^15^,^16^. The use of cps is a very powerful tool to reconstruct plant phylogenies and infer historical biogeographic patterns of diversification^17^,^18^. However, only a limited number of regions in the chloroplast genome have been used to address evolutionary, taxonomic and biodiversity questions in *Cycas*^19^. With the rapid development of Next Generation Sequencing (NGS), it is now feasible to obtain the entire sequence of the chloroplast using a genome skimming approach, resulting in high resolution phylogenies and allowing for estimations of timing of historical diversification, biodiversity and extent of genomic divergence^14^,^20^,^21^.

It is well known that genetic diversity can greatly vary between taxa, due to either different intrinsic characteristics (e.g. reproductive system, genome size and organization) or to extrinsic features (e.g. endemic *vs.* widespread species, young *vs.* old species)^17^,^18^,^22^. In addition, the hypothesis of a linear accumulation of mutations in sequences across time (i.e. a molecular clock) has been refuted in many groups^23^,^24^,^25^.

In this study, we analyse the *C. debaoensis* chloroplast as a reference together with molecular data of other *Cycas* species to identify potential DNA-barcode loci, and compared generic-level chloroplast features in cycads, to highlight the evolutionary history of this group. We also compared the chloroplast features of two individuals of *C. debaoensis* to provide new resources for marker development in this endangered species.

## Results and Discussion

### Genome size and features

**Figure 1:**
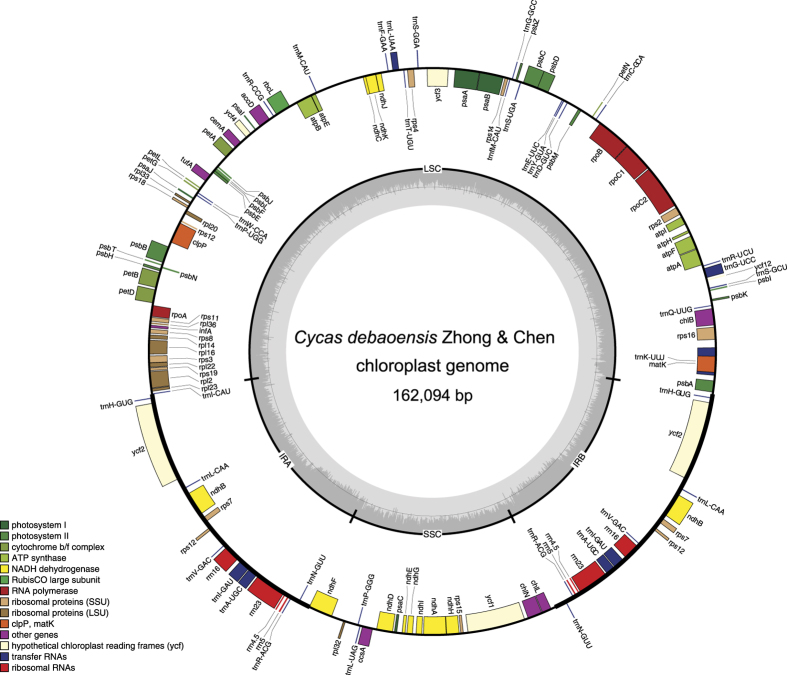
Circular gene map of the plastid genome of *Cycas debaoensis*. Genes drawn within the circle are transcribed clockwise, while those drawn outside are transcribed counter clockwise. Genes are colour-coded according to their functional groups. Inner circle: GC content.

Using genome skimming and reference-guided assembly, we reconstructed the 162,094 bp long chloroplast genome *C. debaoensis*_Jiang_DB-2015. The complete cp genome was submitted to GenBank under accession number KU743927. It was 2 bp longer than *C. debaoensis* (KM459003) due to two 1 bp indels in the LSC (Fig. 1). The two *C. debaoensis* individuals exhibited the typical composition of LSC, SSC regions and two IR copies of 88,854 bp, 23,088 bp, and 25,076 bp (Table 1). The overall GC content of *C. debaoensis*_Jiang_DB-2015 was 39.4%, and 38.7%, 36.6% and 42.0% in the LSC, SSC and IR regions, respectively. These values are similar to *C. debaoensis* KM459003. *C. debaoensis* (KU743927), *C. debaoensis* (KM459003) and *C. revoluta* showed similar GC content (39.4%), slightly lower than *C. taitungensis* (39.5%) (Table 1). In total, 133 genes were annotated, including 87 protein coding genes, 37 tRNA genes and 8 rRNA genes in *C. debaoensis*_Jiang_DB-2015, while 156 and 169 genes were annotated in *C. revoluta* and *C. taitungensis*, respectively. Twelve genes (*atpF, rpoC1*, *rpl2*, *clpP*, *ycf3*, *trnG-UCC*, *trnK-UUU*, *trnL-UAA* in LSC; *ndhA* locates in SSC; *ndhB*, *trnA-UGC*, *trnI-GAU* in the IRs regions) contain 1–2 introns, respectively; while fourteen genes (3 protein coding 7 tRNA and 4 rRNA) were duplicated in the IR regions (Table 2). The *tufA* gene, found in gymnosperms and hornworts and inherited from green algae is coding for a nonfunctional protein synthesis elongation factor (723-bp long in *C. taitungensis* and *Ginkgo*)^17^. Interestingly, this gene was 1 bp longer in *C. debaoensis*_Jiang_DB-2015 than in other *Cycas* (Table 2).

### Repeat and SSR analysis

**Figure 2:**
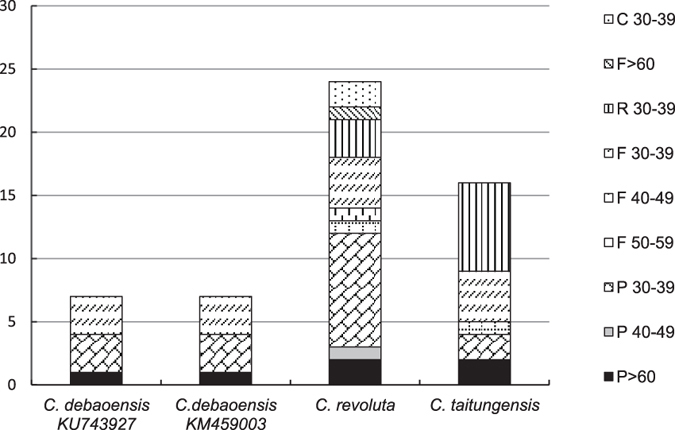
Repeat sequences in four chloroplast genomes of *Cycas*. REPuter was used to identify repeat sequences with length ≥30 bp and sequence identity ≥90% in the chloroplast genomes. F and P indicate the repeat type F (forward) and P (palindrome), respectively. Repeats with different lengths are indicated in different patterns.

**Figure 3:**
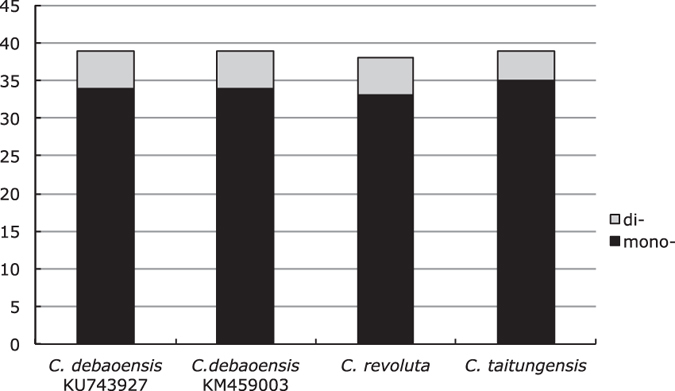
Number of simple sequence repeats in four chloroplast genomes of *Cycas*, classified by repeat type. mono-: mononucleotide SSRs; di-: dinucleotide SSRs.

Using REPuter, seven repeats were found in the chloroplast of *C. debaoensis*_Jiang_DB-2015, which were three forward (F) and four palindrome (P), with no reverse and complement repeats discovered (Table 3). The repeats were mainly distributed in the intergenic spacers of transfer RNA genes, some of them being located in transfer RNA itself (Table 3). Interspecific comparison and analysis in broader *Cycadaceae* showed that *C. revoluta* had the highest number of repeats (24), while *C. debaoensis* contained the fewest (7), and *C. taitungensis* contained an intermediate number of repeats (16) (Supplementary Table S1, Fig. 2). In contrast, the comparison of simple sequence repeats (SSRs) revealed a relative conservatism in their numbers, with congeneric species showing similarities in both numbers and spatial patterns of SSRs occurrence (Fig. 3). This is of particular value in *Cycadaceae*, where species are usually scarcely distributed, and in which diagnostic morphological characters are often poorly defined or absent. These generic molecular biomarkers have the potential to provide useful diagnostic data in redefining complex paraphyletic and polyphyletic species groups in the family^18^,^22^,^26^, aiding directly in conservation efforts.

In other groups, the presence and nature of repeats have been shown to be of great value in evolutionary and population analyses^27^,^28^. Microsatellites (SSRs) are useful markers for population genetics, conservation of endangered species and species delineation^22^,^29^,^30^,^31^, as previously highlighted for *C. debaoensis*^10^,^12^. There were 39 SSRs in the chloroplast of *C. debaoensis*, 34 (87%) and 5 (13%) mono- and di-nucleotides SSRs, respectively (Table 4). These SSRs were mainly distributed in the IGS region (29; 74%), and the other 26% were distributed in CDS genes (Table 4). *C. revoluta* and *C. taitungensis* had similar SSRs numbers and locations than *C. debaoensis*, most of them being mononucleotides and distributed in the IGS (Fig. 3). Interestingly, *C. revoluta* lacked some SSR patterns (G and GA mono- and di- nucleotides, respectively) (Table S2). These diagnostic SSRs can be used in combination with nuclear SSRs developed in the genus for Cycadaceae conservation or reintroduction, species biodiversity assessments and phylogenetic studies in native or introduced areas^12^,^30^,^31^,^32^,^33^.

### Cycads phylogenetic reconstruction and comparison

**Figure 4:**
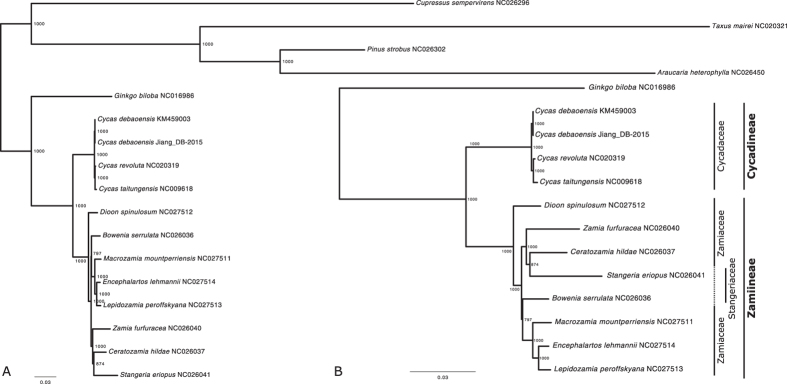
Maximum Likelihood phylogenetic tree of the available chloroplast sequences in GenBank for Cycadales, plus the chloroplast sequences of *Cupressus sempervirens*, *Taxus mairei*, *Pinus strobus*, *Araucaria heterophylla* and *Ginkgo biloba* as outgroups (**A**). For readability and better understanding of the branches lengths, a zoom on the Cycadales family is shown (**B**).

In the maximum likelihood (ML) phylogenetic tree, all but two nodes were highly supported (bootstrap support ≥95), with the accessions of *C. debaoensis* closely related to the other *Cycas* species (Fig. 4A). *Cycas* spp. diverged first in the Cycadales, followed by *Dioon*, a clade containing *Zamia*, *Ceratozamia* and *Stangeria. Bowenia* diverged from the remaining Zamiaceae with relatively high support (bootstrap support 80%), *Macrozamia* being as sister to a clade containing both *Encephalartos* and *Lepidozamia* (Fig. 4A). This chloroplast phylogeny confirms the division of Cycadales into two suborders, each of them being monophyletic in our analyses (Fig. 4B), but contradict the current family delimitations^34^, with the family Stangeriaceae being polyphyletic with high support, in agreement with the most recent phylogenetic work in cycads^35^. However, we found each genus in Stangeriaceae grouping with one of the clade in Zamiaceae, contrary to Salas-Leiva *et al.*^35^, in which *Bowenia* diverged in a basal position for all cycads but *Cycas* and *Dioon*, and *Stangeria* group with *Zamia (Microcycas* being absent from our dataset). *C. debaoensis* diverged in a basal position of the genus, with *C. revoluta* and *C. taitungensis* being closely related. In addition, the branch leading to *Cycas* is longer than the branches leading to the other genera, in accordance with the hypothesis of a recent diversification during the Miocene^19^. These results demonstrate the suitability and efficiency of using complete chloroplast sequences in reconstructing the evolutionary history of cycads, as previously demonstrated for other groups^36^,^37^.

**Figure 5:**
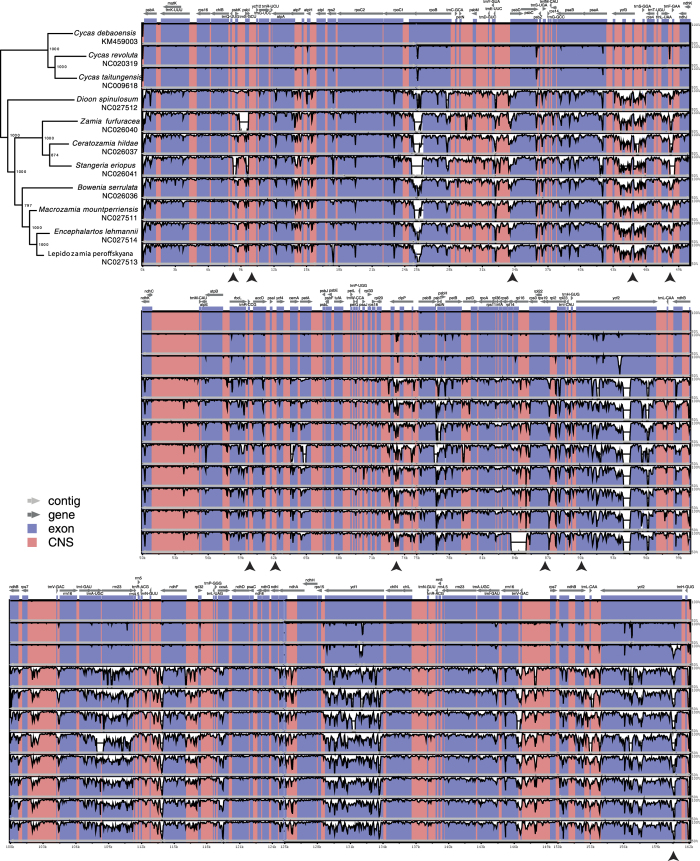
mVISTA percent identity plot of available cycad chloroplasts, using *C. debaoensis* as a reference. Vertical scale indicates the percentage of identity ranging from 50% to 100%. Coding regions are in blue and noncoding regions are in pink. Cladogram redrawn from Fig. 5B; branch lengths are not representative of evolutionary changes; bootstrap support is indicated on the nodes. Black arrows indicate intraspecific variations in *C. debaoensis*.

Although the clear delineation of the genera in cycads are mostly due to the lengths of the sequences provided by complete chloroplast sequencing, the variability was unevenly distributed along the circular molecule (Fig. 5). Indeed, the ribosomal RNA genes as well as the region between *psbA* and *rpoC1* genes (0–25 kb) and between *ndhC* and *rpl20* (50–72 kb) of the cp sequences exhibited relatively few variations among cycads (Fig. 5). A cluster of four *ndh* genes (120–124.5 kb) appeared to be strikingly conserved. Overall, the level of variation increased with taxonomic distance, meaning that regions showing polymorphisms among *Cycas* species, exhibited higher variation among different cycad genera. However, some notable well-defined (<500 bp long) polymorphic regions departed from this assertion in *ycf1* and *ycf2* IRa. Indeed, the polymorphisms in these regions were higher among *Cycas* than among cycads as a whole. Interestingly, three SNPs were located in other regions (*trnL*-*trnF*, *clpP* intron 2 and *ycf2* IRb) that exhibited a continuous increase in polymorphism levels across the family. *ycf1* has been recently proposed as a barcode locus^38^, despite it not being present in all genera^39^, and was identified with *clpP* among the most variable loci in *Parthenium* spp.^40^. Finally, *trnL*-*trnF* was previously used in *Cycas* phylogenetic studies^41^, but also in species identification of trees and ferns^42^,^43^. Here, we stress the need to further assess these loci as potential more informative substitutes to the official barcode loci^44^.

### Comparative interspecific chloroplast genomic analysis

Focusing on the three *Cycas* species available in GenBank, the mVISTA results showed that the four chloroplasts were highly conserved; however, the coding regions appeared to be globally more variable than the non-coding regions (Fig. 5 and Suppl. Fig. 1). Furthermore, the coding regions, e.g. *rpoB*, *psbC*, *clpP* (intron), *ycf1*, and *ycf2*; *psbA*-*trnH* and *trnL*-*trnF* intergenic spacers showed promising levels of variations for further development in applications such as DNA-barcoding or phylogenetic reconstruction.

*Cycas* have been considered as a difficult group for DNA-barcoding^45^. Previously, it was reported that the *psbA*-*trnH* spacer was highly variable in Cycadales except *Cycas*^45^, but *trnL*-*trnF* was used in *Cycas* for phylogenetic studies^41^. Although *rbcL* + *matK* were chosen as a two-locus DNA-barcode for their universality and efficacy in land plant^44^, it was not variable enough in *Cycas* (Fig. 5 and Suppl. Fig. 1). *rpoB*, *rpoC1*, and non-coding (e.g. *atpF*-*atpH, psbK*-*psbI*, and *rpl32*-*trnL*) regions have been shown to be variable enough at higher taxonomic levels^46^, but also within cycads^47^,^48^. In light of this, *psbC*, *clpP* (intron), *ycf1*, and *ycf2* should be considered as candidates for future phylogenetic studies in *Cycas*.

### Intraspecific comparison

Comparing the two individuals of *C. debaoensis*, we found 10 SNPs and 1 indel, plus one “N” position in our data that didn’t allow us to confirm its status (Table 5). Their genomic positions are indicated in Figs 1 and 5. Six and four SNPs were located in IGS and coding regions, respectively, and one indel in the *clpC* intron. The genetic distance between the two individuals was 0.005553% (i.e. ≈1 SNPs/indels per 20 kb). This result is consistent with previous studies showing low within-population diversity in addition to limited gene flow among populations in *C. debaoensis*^10^,^11^. Whittall *et al.*^49^ found a comparable level of intraspecific divergence in pines, irrespective of the rarity of the considered species. However, in the pest species *Jacobaea vulgaris* (Asteraceae), the intraspecific divergence was four times higher^50^, perhaps due to it’s short generation times as opposed to those prevailing in slow growing and long-lived trees. Further studies are still needed to determine whether or not intraspecific genetic diversity is linked to geographic ranges or the intrinsic characteristics of the taxonomic group.

## Conclusions

Comparing genomic diversity at different taxonomical, but also spatial and temporal scales, we were able to reconstruct a robust phylogeny for cycads, and to identify regions showing promising levels of variation at three levels (familial, generic and intraspecific rank). These regions can provide useful and alternative loci for species identification and population-based studies for conservation, ecology and evolution. Despite their restricted geographic ranges, we showed that several, potentially diagnostic intraspecific variations can be found in the chloroplasts of different individuals *C. debaoensis*, including 10 SNPs and 1 indel in as of yet unstudied regions. Comparing results from the three scales, four regions appeared to be variable at the three considered taxonomic scales, namely *ycf3*, *clpP*, *psbD* and the *trnL-trnF* IGS. Therefore, we recommend future studies in cycads further evaluate these loci in details.

We expect that by providing and highlighting these new resources to the plant research community, it will allow for development of new diagnostic markers and innovative conservation strategies in this iconic, but highly threatened taxonomic group, especially in the case of *C. debaoensis*.

## Materials and Methods

### DNA sequencing and genome assembly

Total genomic DNA was extracted from 0.1 g of frozen fresh leaves, from an individual collected in Guangxi (23°69′40″N, 106°15′83″E) in 2015 (voucher deposited at our research group herbarium, Jiang_C2) according to the manufacturer instructions with the Plant Genomic DNA Kit (Tiangen Biotech Co., Ltd). A 350-bp paired-end library was then constructed using NEBNext Ultra II DNA Library Prep Kit (Ipswich, Massachusetts, USA) and sequenced by Novogene (Beijing, China). About 1 Gb of raw data were obtained on an Illumina HiSeq2500 platform (San Diego, California, USA), with a paired-end read length of 2 × 150 bp. The raw reads were submitted to the SRA under the accession number SRR3407155.

The raw data were imported in Geneious R9 (Biomatters Ltd, Auckland, New Zealand), and a cp genome was assembled according to Hinsinger and Strijk^51^. Raw reads were trimmed according to their 5′ and 3′ -end quality, then a reference-guided assembly was performed, using the available cp of *Cycas debaoensis* (KM459003) as a reference for the mapping step. The cp genome annotation was transferred from *C. debaoensis* using the implemented function in Geneious R9 and their boundaries were manually checked. The circular cp genome map was generated using the Organellar Genome Draw program (OGDRAW).

### Intergeneric comparisons and phylogenetic reconstruction

Following the recommended best practices for complete organellar sequencing^52^, we performed a phylogenetic analysis to confirm the accuracy of our reconstructed plastid and sample identification. We retrieved all the Cycadales available in GenBank (accessed 2016/02/15), representing all the ten genera in the order except *Microcycas*^6^. To this Cycadales dataset, we added the sequences of *Gingko biloba* (NC016986), *Pinus strobus* (NC026302), *Araucaria heterophylla* (NC026450), *Taxus mairei* (NC020321) and *Cupressus sempervirens* (NC026296) as outgroups.

Sequences were aligned using MAFFT^53^ with default options. We used the jModelTest^54^ implementation in CIPRES^55^, and set the substitution model accordingly. We built a maximum likelihood tree using PHYML^56^, with a 012310 + G + F model using four gamma categories and 1000 bootstrap replicates. The ML tree was built using all positions. In addition, to identify regions with substantial variability, the complete cp genomes of nine cycad genera were compared using mVISTA^57^, with *C. debaoensis* (KM459003) as a reference for the annotations.

Sequence divergences among cycads were estimated using the Kimura 2-parameter model^58^, implemented in MEGA6^59^. Codon positions included were 1st + 2nd + 3rd + Noncoding. All positions containing gaps and missing data were excluded prior to analyses.

### Interspecific comparisons

The complete cp genomes of *C. debaoensis* and two other species in cycas (*C. revoluta* and *C. taitungensis*) were compared using mVISTA^57^, as described above.

For each species, repeats (forward, palindrome, reverse and complement sequences) were identified using REPuter^60^ with 30 bp and sequence identity greater than 90%. Simple sequence repeats (SSRs) of *C. debaoensis* and the two other species were detected using MISA^61^ by setting the minimum number of repeats to 10, 5, 4, 3, 3 and 3 for mono-, di-, tri-, tetra-, penta- and hexa nucleotides, respectively. Sequence divergences among the *Cycas* species were estimated as described above.

### Intraspecific genome comparison

The two complete chloroplasts of *C. debaoensis* (KU743927, KM459003) were aligned in Geneious R9 (Biomatters Ltd, Auckland, New Zealand) using the MAFFT algorithm^53^, and differences were identified using the “Find Variations/SNPs” function and checked individually. We recorded substitutions and indels separately, as well as their location in the chloroplast genome (e.g. SSRs/repeats, coding region/rRNA/tRNA/IGS). Sequence divergence extent between the two individuals was estimated as described above.

## Additional Information

**Accession codes**: Raw reads and assembled chloroplast of C. debaoensis are available under the accession numbers SRR3407155 and KU743927, respectively.

**How to cite this article**: Jiang, G.-F. *et al.* Comparison of intraspecific, interspecific and intergeneric chloroplast diversity in Cycads. *Sci. Rep.* **6**, 31473; doi: 10.1038/srep31473 (2016).

## Supplementary Material

Supplementary Information

## Figures and Tables

**Table 1:** Characteristics of the complete chloroplasts used in the study.

Species	Length	LSC	SSC	IR	GC%				Coding	tRNA	rRNA
					Total	LSC	SSC	IR			
*C. debaoensis* KU743927	162,094	88,854	23,088	25,076	39.4	38.7	36.6	42.0	87	37	8
*C. debaoensis*	162,092	88,852	23,088	25,076	39.4	38.7	36.5	42.0	87	37	8
*C. revoluta*	162,489	88,977	23,376	25,068	39.4	38.6	38.9	42.0	109	39	8
*C. taitungensis*	163,403	90,216	23,039	25,074	39.5	38.8	36.5	42.0	122	38	8

The Length, Large Single Copy (LSC) region; Small Single Copy (SSC) region; Inverted Repeats (IR), the GC content of each region, and the number of coding, tRNA and rRNA loci are shown. Data from GenBank for *C. debaoensis* (KM459003), *C. taitungensis*^17^ (NC_009618) and *C. revoluta* (NC_020319).

**Table 2:** Genes present in the *C.debaoensis* (KU743927) chloroplast genome.

	Group of genes	Gene names
1	Photosystem I	*psaA*, *psaB*, *psaC*, *psaI*, *psaJ*
2	Photosystem II	*psbA*, *psbB*, *psbC*, *psbD*, *psbE*, *psbF*, *psbH*, *psbI*, *psbJ*, *psbK*, *psbL*, *psbM*, *psbN*, *psbT*, *psbZ*
3	Cytochrome b/f complex	*petA*, *petB*, *petD*, *petG*, *petL*, *petN*
4	ATP synthase	*atpA*, *atpB*, *atpE*, *atpF*^a^, *atpH*, *atpI*
5	NADH dehydrogenase	*ndhA*^a^, *ndhB*^a^ (×2), *ndhC*, *ndhD*, *ndhE*, *ndhF*, *ndhG*, *ndhH*, *ndhI*, *ndhJ*, *ndhK*
6	RubisCO large subunit	*rbcL*
7	RNA polymerase	*rpoA*, *rpoB*, *rpoC1*^a^, *rpoC2*
8	Ribosomal proteins (SSC)	*rps2*, *rps3*, *rps4*, *rps7* (×2), *rps8*, *rps11*, *rps12* (×2), *rps14*, *rps15*, *rps16*, *rps18*, *rps19*
9	Ribosomal proteins (LSC)	*rpl2* a, *rpl14*, *rpl16*, *rpl20*, *rpl22*, *rpl23*, *rpl32*, *rpl33*, *rpl36*
10	Other genes	*accD*, *ccsA*, *cemA*, *chlB*, *chlL*, *chlN*, *clpP*^b^, *infA*, *matK, tufa*
11	Proteins of unknown function	*ycf1*, *ycf2* (×2), *ycf3*^b^, *ycf4*, *ycf12*
12	Ribosomal RNAs	*rrn4.5* (×2), *rrn5* (×2), *rrn16* (×2), *rrn23* (×2)
13	Transfer RNAs	*trnA-UGC*^a^ (×2), *trnC-GCA*, *trnD-GUC*, *trnE-UUC*, *trnF-GAA*, *trnfM-CAU*, *trnG-GCC*, *trnG-UCC*^a^, *trnH-GUG* (×2), *trnI-CAU*, *trnI-GAU*^a^ (×2), *trnK-UUU*^a^, *trnL-CAA* (×2), *trnL-UAA*^a^, *trnL-UAG*, *trnM-CAU*, *trnN-GUU* (×2), *trnP-GGG*, *trnP-UGG*, *trnQ-UUG*, *trnR-ACG* (×2), *trnR-CCG*, *trnR-UCU*, *trnS-GCU*, *trnS-GGA*, *trnS-UGA*, *trnT-UGU*, *trnV-GAC* (×2), *trnW-CCA*, *trnY-GUA*

(×2) Two gene copies in the IRs.

^a^Gene containing one intron.

^b^Gene containing two introns.

**Table 3:** Repeat sequences and their distribution found by REPuter in the *C. debaoensis* (KU743927) chloroplast genome.

	Repeat1 start (location)	Repeat2 start (location)	Size (bp)	Type	Region
1	88,852 (*trnI-CAU*)	137,037 (IGS *chlL*-*trnN-GUU*)	20,257	P	LSC; IRb
2	28,396 (IGS *ropB*-*trnC-GCA*)	28,470 (IGS *ropB*-*trnC-GCA*)	39	F	LSC
3	55,293 (*trnM-CAU*)	55,340 (IGS *trnM-CAU*-*atpE*)	32	P	LSC
4	48,603 (IGS *trnF-GAA-ndhJ*)	48,629 (IGS *trnF-GAA-ndhJ*)	31	F	LSC
5	113,680 (IGS *trnN-GUU-ndhF*)	113,680 (IGS *trnN-GUU-ndhF*)	30	P	IRa
6	113,680 (IGS *trnN-GUU-ndhF*)	137,236 (IGS *chlL-trnN-GUU*)	30	F	IRa; IRb
7	137,236 (IGS *chlL-trnN-GUU*)	137,236 (IGS *chlL-trnN-GUU*)	30	P	IRb

IGS: Intergenic spacer.

**Table 4:** Simple sequence repeats in the *C. debaoensis* (KU743927) chloroplast genome.

No.	SSR type	SSR	size	start	SSR-containing region
2	p1	A	20	8307	IGS (*trnQ-UUG*-*psbK*); LSC
	p1	A	20	73073	*clpP*; LSC
1	p1	A	19	123912	IGS (*ndhG*-*ndhI*); SSC
1	p1	A	14	32963	IGS (*trnE-UUC*-*trnH-GUG*); LSC
1	p1	A	13	68694	IGS (*tufA*-*trnH-GUG*); LSC
2	p1	A	11	101911	IGS (*rps7*-*rps12*); IRa
	p1	A	11	148078	IGS (*trnV-GAC*-*rps12*); IRb
2	p1	A	10	1872	*trnK-UUU*; LSC
	p1	A	10	11723	IGS (*trnG-UCC*-*trnG-UCC*); LSC
1	p1	T	19	15487	IGS (*atpH*-*atpI*); LSC
1	p1	T	18	10133	IGS (*trnS-GCU*-*ycf12*); LSC
2	p1	T	16	57648	IGS (*atpB*-*rbcL*); LSC
	p1	T	16	84489	*rpl16*; LSC
3	p1	T	15	54643	IGS (*trnM-CAU*-*ndhC*); LSC
	p1	T	15	84795	*rpl16*; LSC
	p1	T	15	134345	IGS (*ycf1*-*chlN*); SSC
1	p1	T	13	74064	*clpP*; LSC
2	p1	T	12	5951	*rps16*; LSC
	p1	T	12	118369	IGS (*rpl32*-*trnP-GGG*); SSC
4	p1	T	11	83227	*rps8*; LSC
	p1	T	11	88618	IGS (*rpl23*-*trnI-CAU*); LSC
	p1	T	11	102859	IGS (*rps12*-*trnV-GAC*); IRa
	p1	T	11	149026	IGS (*rps7*-*rps12*);IRb
3	p1	T	10	63350	IGS (*ycf4*-*cemA*); LSC
	p1	T	10	69919	IGS (*trnP-UGG*-*psaJ*); LSC
	p1	T	10	125905	*ndhA*; SSC
1	p1	G	14	70451	IGS (*psaJ-trnH-GUG*); LSC
1	p1	G	11	52764	IGS (*ndhC-trnH-GUG*); LSC
2	p1	G	10	70921	IGS (*rpl33-trnH-GUG*); LSC
	p1	G	10	149995	IGS (*rps7-ndhB*); IRb
1	p1	C	14	44747	*ycf3*; LSC
1	p1	C	11	5229	*rps16*; LSC
2	p1	C	10	17681	IGS (*rps2-rpoC2*); LSC
	p1	C	10	100943	IGS (*ndhB-rps7*); IRa
3	p2	(TA)14	28	29832	IGS (*petN-psbM*); LSC
	p2	(TA)9	18	1453	IGS (*psbA-trnK-UUU*); LSC
	p2	(TA)6	12	15670	IGS (*atpH-atpI*); LSC
1	p2	(GA)6	12	68377	IGS (*psbE-petL*); LSC
1	p2	(AT)6	12	15808	IGS (*atpH-atpI*); LSC

**Table 5:** Intraspecific SNPs between two individuals of *C. debaoensis* (KU743927).

Position	Type	Nucleotide	location	location type
8432		A/T	*trnQ*-*psbK*	IGS
9889		C/T	*trnS*-*ycf12*	IGS
33707		G/T	*psbD*	coding
44878		C/G	*ycf3*	exon 1
48140		A/C	*trnL*-*trnF*	IGS
60132		G/T	*trnR*-*accD*	IGS
62218		A/C	*psaI*-*ycf4*	IGS
73073		A/.	*clpP*	intron 2
86731		C/T	*rps19*-*rpl2*	IGS
90307		G/T	*ycf2*	coding (IRa)
160640		A/C	*ycf2*	coding (IRb)

## References

[b1] Brenner E. D., Stevenson D. W. & Twigg R. W. Cycads: evolutionary innovations and the role of plant-derived neurotoxins. Trends Plant Sci. 8, 446–452 (2003). 13678912 10.1016/S1360-1385(03)00190-0

[b2] Mamay S. H. Cycads: fossil evidence of late paleozoic origin. Science. 164, 295–296 (1969). 17812085 10.1126/science.164.3877.295

[b3] Gao Z. & Thomas B. A. A review of fossil cycad megasporophylls, with new evidence of Crossozamia Pomel and its associated leaves from the Lower Permian of Taiyuan, China. Rev. Palaeobot. Palynol. 60, 205–223 (1989).

[b4] Nagalingum N. *et al.* Recent synchronous radiation of a living fossil. Science. 334, 796–799 (2011). 22021670 10.1126/science.1209926

[b5] Hill K. D., Stevenson D. W. & Osborne R. The world list of cycads. Bot. Rev. 70, 274–298 (2004).

[b6] Calonje M., Stevenson D. W. & Stanberg L. The World List of Cycads. *Online edition* [*Internet*], Available from: http://www.cycad list.org. (2013–2016).

[b7] Marler P. N. & Marler T. E. An assessment of Red List data for the Cycadales. Trop. Conserv. Sci. 8 (2015).

[b8] Chen C. J. & Liu N. New discoveries of cycads and advancement of conservation of cycads in China. Bot. Rev. 70, 93–100 (2004).

[b9] Chen C. J. & Zhong Y. C. *Cycas debaoensis* Y. C. Zhong et C. J. Chen– a new cycad from China. Acta Phytotaxon. Sin. 35, 571 (1997).

[b10] Zhan Q.-Q., Wang J.-F., Gong X. & Peng H. Patterns of chloroplast DNA variation in *Cycas debaoensis* (Cycadaceae): conservation implications. Conserv. Genet. 12, 959–970 (2011).

[b11] Xie J., Jian S. & Liu N. Genetic variation in the endemic plant *Cycas debaoensis* on the basis of ISSR analysis. Aust. J. Bot. 53, 141–145 (2005).

[b12] Yang Y., Li Y., Li L.-F., Ge X.-J. & Gong X. Isolation and characterization of microsatellite markers for *Cycas debaoensis* YC Zhong et CJ Chen (Cycadaceae). Mol. Ecol. Resour. 8, 913–915 (2008). 21585928 10.1111/j.1755-0998.2008.02114.x

[b13] Jansen R. K. & Ruhlman T. A. Plastid genomes of seed plants. InGenomics of chloroplasts and mitochondria. 103–126 (Springer, 2012).

[b14] Staats M. *et al.* Genomic treasure troves: complete genome sequencing of herbarium and insect museum specimens. PLoS One. 8, e69189 (2013). 23922691 10.1371/journal.pone.0069189 PMC3726723

[b15] Nock C. J. *et al.* Chloroplast genome sequences from total DNA for plant identification. Plant Biotechnol. J. 9, 328–333 (2011). 20796245 10.1111/j.1467-7652.2010.00558.x

[b16] Coissac E., Hollingsworth P. M., Lavergne S. & Taberlet P. From barcodes to genomes: extending the concept of DNA barcoding. Mol. Ecol. (2016). 10.1111/mec.13549 26821259

[b17] Wu C.-S., Wang Y.-N., Liu S.-M. & Chaw S.-M. Chloroplast genome (cpDNA) of *Cycas taitungensis* and 56 cp protein-coding genes of *Gnetum parvifolium*: insights into cpDNA evolution and phylogeny of extant seed plants. Mol. Biol. Evol. 24, 1366–1379 (2007). 17383970 10.1093/molbev/msm059

[b18] Jardón-Barbolla L., Delgado-Valerio P., Geada-López G., Vázquez-Lobo A. & Piñero D. Phylogeography of *Pinus* subsection Australes in the Caribbean basin. Ann. Bot. mcq232 (2010). 10.1093/aob/mcq232 PMC3025731 21118838

[b19] Xiao L.-Q. & Möller M. Nuclear ribosomal its functional paralogs resolve the phylogenetic relationships of a late-Miocene radiation cycad *Cycas* (Cycadaceae). PLoS One. 10, e0117971 (2015). 25635842 10.1371/journal.pone.0117971 PMC4311995

[b20] Straub S. C. *et al.* Navigating the tip of the genomic iceberg: Next-generation sequencing for plant systematics. Am. J. Bot. 99, 349–364 (2012). 22174336 10.3732/ajb.1100335

[b21] Hinsinger D. D. & Strijk J. S. Complete chloroplast genome sequence of *Castanopsis concinna* (Fagaceae), a threatened species from Hong Kong and South-Eastern China. Mitochondrial DNA. 1–2 (2015). 10.3109/19401736.2015.1110800 26678387

[b22] Wu C.-S., Lin C.-P., Hsu C.-Y., Wang R.-J. & Chaw S.-M. Comparative chloroplast genomes of Pinaceae: insights into the mechanism of diversified genomic organizations. Genome Biol. Evol. 3, 309–319 (2011). 21402866 10.1093/gbe/evr026 PMC5654405

[b23] Bell C. D., Soltis D. E. & Soltis P. S. The age of the angiosperms: a molecular timescale without a clock. Evolution. 59, 1245–1258 (2005). 16050101

[b24] Kay K. M., Whittall J. B. & Hodges S. A. A survey of nuclear ribosomal internal transcribed spacer substitution rates across angiosperms: an approximate molecular clock with life history effects. BMC Evol. Biol. 6, 36 (2006). 16638138 10.1186/1471-2148-6-36 PMC1484492

[b25] Pereira S. L. & Baker A. J. A mitogenomic timescale for birds detects variable phylogenetic rates of molecular evolution and refutes the standard molecular clock. Mol. Biol. Evol. 23, 1731–1740 (2006). 16774978 10.1093/molbev/msl038

[b26] Lin C.-P., Wu C.-S., Huang Y.-Y. & Chaw S.-M. The complete chloroplast genome of *Ginkgo biloba* reveals the mechanism of inverted repeat contraction. Genome Biol. Evol. 4, 374–381 (2012). 22403032 10.1093/gbe/evs021 PMC3318433

[b27] Cavalier-Smith T. Chloroplast evolution: secondary symbiogenesis and multiple losses. Curr. Biol. 12, R62–R64 (2002). 11818081 10.1016/s0960-9822(01)00675-3

[b28] Nie X. *et al.* Complete chloroplast genome sequence of a major invasive species, crofton weed (*Ageratina adenophora*). PLoS One. 7, e36869 (2012). 22606302 10.1371/journal.pone.0036869 PMC3350484

[b29] Powell W., Morgante M., McDevitt R., Vendramin G. & Rafalski J. Polymorphic simple sequence repeat regions in chloroplast genomes: applications to the population genetics of pines. Proc. Natl. Acad. Sci. USA 92, 7759–7763 (1995). 7644491 10.1073/pnas.92.17.7759 PMC41225

[b30] Cibrián-Jaramillo A., Marler T. E., DeSalle R. & Brenner E. D. Development of EST-microsatellites from the cycad *Cycas rumphii*, and their use in the recently endangered *Cycas micronesica*. Conserv. Genet. 9, 1051–1054 (2008).

[b31] Calonje M. *et al.* Cycad biodiversity in the Bahamas Archipelago and conservation genetics of the threatened *Zamia lucayana* (Zamiaceae). Oryx. 47, 190–198 (2013).

[b32] Cibrián-Jaramillo A., Daly A., Brenner E., Desalle R. & Marler T. When North and South don’t mix: genetic connectivity of a recently endangered oceanic cycad, *Cycas micronesica*, in Guam using EST-microsatellites. Mol. Ecol. 19, 2364–2379 (2010). 20497328 10.1111/j.1365-294X.2010.04638.x

[b33] Ju L.-P. *et al.* Microsatellite primers in the native perennial cycad *Cycas taitungensis* (Cycadaceae). Am. J. Bot. 98, e84–e86 (2011). 21613154 10.3732/ajb.1000504

[b34] Stevenson D. W. A formal classification of the extant cycads. Brittonia. 44, 220–223 (1992).

[b35] Salas-Leiva D. E. *et al.* Phylogeny of the cycads based on multiple single-copy nuclear genes: congruence of concatenated parsimony, likelihood and species tree inference methods. Ann. Bot. 112, 1263–1278 (2013). 23997230 10.1093/aob/mct192 PMC3806525

[b36] Jansen R. K. *et al.* Phylogenetic analyses of *Vitis* (Vitaceae) based on complete chloroplast genome sequences: effects of taxon sampling and phylogenetic methods on resolving relationships among rosids. BMC Evol. Biol. 6, 1 (2006). 16603088 10.1186/1471-2148-6-32 PMC1479384

[b37] Parks M., Cronn R. & Liston A. Increasing phylogenetic resolution at low taxonomic levels using massively parallel sequencing of chloroplast genomes. BMC Boil. 7, 1 (2009). 10.1186/1741-7007-7-84 PMC2793254 19954512

[b38] Dong W. *et al.* *ycf1*, the most promising plastid DNA barcode of land plants. Sci. Rep. 5 (2015). 10.1038/srep08348 PMC4325322 25672218

[b39] Dong W., Liu J., Yu J., Wang L. & Zhou S. Highly variable chloroplast markers for evaluating plant phylogeny at low taxonomic levels and for DNA barcoding. PLoS One. 7, e35071 (2012). 22511980 10.1371/journal.pone.0035071 PMC3325284

[b40] Kumar S., Hahn F. M., McMahan C. M., Cornish K. & Whalen M. C. Comparative analysis of the complete sequence of the plastid genome of Parthenium argentatum and identification of DNA barcodes to differentiate *Parthenium* species and lines. BMC Plant Biol. 9, 1 (2009). 10.1186/1471-2229-9-131 PMC2784773 19917140

[b41] Sangin P., Lindstrom A. J., Kokubugata G., Chaiprasongsuk M. & Mingmuang M. Phylogenetic relationships within Cycadaceae inferred from non-coding regions of chloroplast DNA. J. Nat. Sci. 44, 544–557 (2010).

[b42] Kojoma M. *et al.* Genetic identification of cinnamon (Cinnamomum spp.) based on the *trnL*-*trnF* chloroplast DNA. Planta Medica. 68, 94–96 (2002). 11842343 10.1055/s-2002-20051

[b43] De Groot G. A. *et al.* Use of *rbcL* and *trnL*-F as a two-locus DNA barcode for identification of NW-European ferns: an ecological perspective. PLoS One. 6, e16371 (2011). 21298108 10.1371/journal.pone.0016371 PMC3027654

[b44] CBOL Plant Working Group. A DNA barcode for land plants. Proc. Natl. Acad. Sci. USA 106, 12794–12797 (2009). 19666622 10.1073/pnas.0905845106 PMC2722355

[b45] Sass C., Little D. P., Stevenson D. W. & Specht C. D. DNA barcoding in the cycadales: testing the potential of proposed barcoding markers for species identification of cycads. PLoS One. 2, e1154 (2007). 17987130 10.1371/journal.pone.0001154 PMC2063462

[b46] Li X. *et al.* Plant DNA barcoding: from gene to genome. Biol. Rev. 90, 157–166 (2015). 24666563 10.1111/brv.12104

[b47] Nicolalde-Morejón F., Vergara-Silva F., González-Astorga J. & Stevenson D. W. Character-based, population-level DNA barcoding in Mexican species of *Zamia* L. (Zamiaceae: Cycadales). Mitochondrial DNA. 21, 51–59 (2010). 21271859 10.3109/19401736.2010.539215

[b48] Nicolalde-Morejón F. *et al.* A character-based approach in the Mexican cycads supports diverse multigene combinations for DNA barcoding. Cladistics. 27, 150–164 (2011). 10.1111/j.1096-0031.2010.00321.x 34875777

[b49] Whittall J. B. *et al.* Finding a (pine) needle in a haystack: chloroplast genome sequence divergence in rare and widespread pines. Mol. Ecol. 19, 100–114 (2010). 20331774 10.1111/j.1365-294X.2009.04474.x

[b50] Doorduin L. *et al.* The complete chloroplast genome of 17 individuals of pest species *Jacobaea vulgaris*: SNPs, microsatellites and barcoding markers for population and phylogenetic studies. DNA Res. dsr002 (2011). 10.1093/dnares/dsr002 PMC3077038 21444340

[b51] Hinsinger D. D. & Strijk J. S. Toward phylogenomics of Lauraceae: The complete chloroplast genome sequence of *Litsea glutinosa* (Lauraceae), an invasive tree species on Indian and Pacific Ocean islands. *Plant Gene*, submitted.

[b52] Botero-Castro F., Delsuc F. & Douzery E. J. Thrice better than once: Quality control guidelines to validate new mitogenomes. Mitochondrial DNA. 27, 449–454 (2016). 24708133 10.3109/19401736.2014.900666

[b53] Katoh K. & Standley D. M. MAFFT multiple sequence alignment software version 7: improvements in performance and usability. Mol. Biol. Evol. 30, 772–780 (2013). 23329690 10.1093/molbev/mst010 PMC3603318

[b54] Darriba D., Taboada G. L., Doallo R. & Posada D. jModelTest 2: more models, new heuristics and parallel computing. Nat. Methods. 9, 772–772 (2012). 22847109 10.1038/nmeth.2109 PMC4594756

[b55] Miller M. A., Pfeiffer W. & Schwartz T. Creating the CIPRES Science Gateway for inference of large phylogenetic trees. In Gateway Computing Environments Workshop (GCE), 1–8 (IEEE) (2010).

[b56] Guindon S., Delsuc F., Dufayard J.-F. & Gascuel O. Estimating maximum likelihood phylogenies with PhyML. Methods Mol. Biol. 537, 113–137 (2009). 19378142 10.1007/978-1-59745-251-9_6

[b57] Frazer K. A., Pachter L., Poliakov A., Rubin E. M. & Dubchak I. VISTA: computational tools for comparative genomics. Nucleic Acids Res. 32, W273–W279 (2004). 15215394 10.1093/nar/gkh458 PMC441596

[b58] Kimura M. A simple method for estimating evolutionary rates of base substitutions through comparative studies of nucleotide sequences. J. Mol. Evol. 16, 111–120 (1980). 7463489 10.1007/BF01731581

[b59] Tamura K., Stecher G., Peterson D., Filipski A. & Kumar S. MEGA6: molecular evolutionary genetics analysis version 6.0. Mol. Biol. Evol. mst197 (2013). 10.1093/molbev/mst197 PMC3840312 24132122

[b60] Kurtz S. & Schleiermacher C. REPuter: fast computation of maximal repeats in complete genomes. Bioinformatics. 15, 426–427 (1999). 10366664 10.1093/bioinformatics/15.5.426

[b61] Thiel T., Michalek W., Varshney R. & Graner A. Exploiting EST databases for the development and characterization of gene-derived SSR-markers in barley (*Hordeum vulgare* L.). Theor. Appl. Genet. 106, 411–422 (2003). 12589540 10.1007/s00122-002-1031-0

