# Supplementary material for: Comparison of intraspecific, interspecific and intergeneric chloroplast diversity in Cycads
Source: Sci Rep. 2016 Aug 25;6:31473. doi: 10.1038/srep31473 (PMC4997344; doi:10.1038/srep31473)
Supplement: Supplementary Information [file srep31473-s1.pdf]

Comparison of intraspecific, interspecific and intergeneric chloroplast diversity in Cycads

Guo-Feng Jiang[1†*]; Damien Daniel Hinsinger[1†]; Joeri Sergej Strijk[1]

[1] Plant Ecophysiology and Evolution Group, State Key Laboratory for Conservation and Utilization of Subtropical Agro-Bioresources and College of Forestry, Guangxi University, Nanning, Guangxi 530004, China;

† Authors contributed equally

[*] Corresponding author (gfjiang@gxu.edu.cn)

Supplementary material

Supplementary Table S1. Repeat sequences and their distribution found by REPuter in the *C. debaoensis* (KM459003), *C. revoluta*, and *C. taitungensis* chloroplast genomes. IGS: Intergenic spacer.

Supplementary Table S2. Simple sequence repeats in the *C. debaoensis* (KM459003), *C. revoluta*, and *C. taitungensis* chloroplast genomes. IGS: Intergenic spacer.

Supplementary Figure 1. mVISTA percent identity plot comparing the four *Cycas* chloroplast genomes with *C. debaoensis* as a reference. Vertical scale indicates the percentage of identity ranging from 50% to 100%. Coding regions are in blue and noncoding regions are in pink.

Table S1. Repeat sequences and their distribution found by REPuter in the *C. debaoensis* KM459003, *C. revoluta*, and *C. taitungensis* chloroplast genome. IGS: Intergenic spacer.

	Repeat1 start (location)	Repeat2 start (location)	Size(bp)	Type	Region
***C. debaoensis* KM459003**					
1	88,850 (*trnI-CAU*)	137,035 (IGS *chlL-trnN-GUU*)	20,257	P	LSC; IRb
2	28,396 (IGS *ropB-trnC-GCA*)	28,470 (IGS *ropB-trnC-GCA*)	39	F	LSC
3	55,292 (*trnM-CAU*)	55,339 (IGS *trnM-CAU-atpE*)	32	P	LSC
4	48,602 (IGS *trnF-GAA-ndhJ*)	48,628 (IGS *trnF-GAA-ndhJ*)	31	F	LSC
5	113,678 (IGS *trnN-GUU-ndhF*)	113,678 (IGS *trnN-GUU-ndhF*)	30	P	IRa
6	113,678 (IGS *trnN-GUU-ndhF*)	137,234 (IGS *chlL-trnN-GUU*)	30	F	IRa; IRb
7	137,234 (IGS *chlL-trnN-GUU*)	137,234 (IGS *chlL-trnN-GUU*)	30	P	IRb
C. revoluta					
1	0 (IGS *ndhF -trnA-AAC*)	114,044 (IGS *psbA-tRNA-CAC*)	25,066	P	LSC; IRa
2	0 (IGS *ndhF -trnA-AAC*)	162,145 (*ndhF*)	280	F	SSC
3	138,830 (IGS *trnA-AAC - chIL*)	162,145 (*ndhF*)	280	P	IRb; SSC
4	85,513 (IGS *trnA-UGC-rpoB*)	85,587 (IGS *trnA-UGC-rpoB*)	55	F	LSC
5	282 (IGS *ndhF -tRNA-AAC*)	162,427 (IGS *ndhF -trnA-AAC*)	46	F	IRa; SSC
6	138,782(IGS *trnA-AAC -chIL*)	162,427 (IGS *ndhF -trnA-AAC*)	46	P	IRb; SSC
7	84,159 (IGS *psbM-petN*)	84,159 (IGS *psbM-petN*)	33	R	LSC; LSC
8	58,625 (IGS *atpE-tRNA-AUG*)	58,672 (IGS *atpE-tRNA-AUG*)	32	P	LSC; LSC
9	84,159 (IGS *psbM-petN*)	84,159 (IGS *psbM-petN*)	32	P	LSC; LSC
10	84,159 (IGS *psbM-petN*)	84,160 (IGS *psbM-petN*)	32	C	LSC; LSC
11	84,160 (IGS *psbM-petN*)	84,160 (IGS *psbM-petN*)	32	P	LSC; LSC
12	65,357 (IGS *ndhJ-tRNA-UUC*)	65,383 (IGS *ndhJ-tRNA-UUC*)	31	F	LSC; LSC
13	84,159 (IGS *psbM-petN*)	84,159 (IGS *psbM-petN*)	31	R	LSC; LSC
14	84,159 (IGS *psbM-petN*)	84,161 (IGS *psbM-petN*)	31	F	LSC; LSC
15	84,161(IGS *psbM-petN*)	84,161(IGS *psbM-petN*)	31	R	LSC; LSC
16	218 (IGS *ndhF -tRNA-AAC*)	218 (IGS *ndhF -tRNA-AAC*)	30	P	IRa; IRa
17	218 (IGS *ndhF -tRNA-AAC*)	138,862 (IGS *trnA-AAC-chIL*)	30	F	IRa; IRb
18	218 (IGS *ndhF -tRNA-AAC*)	162,363 (IGS *ndhF -trnA-AAC*)	30	P	IRa; SSC
19	84,159 (IGS *psbM-petN*)	84,159 (IGS *psbM-petN*)	30	P	LSC; LSC
20	84,159 (IGS *psbM-petN*)	84,162 (IGS *psbM-petN*)	30	C	LSC; LSC
21	84,162 (IGS *psbM-petN*)	84,162 (IGS *psbM-petN*)	30	P	LSC; LSC
22	138, 862 (IGS *trnA-AAC-chIL*)	138,862 (IGS *trnA-AAC-chIL*)	30	P	IRb; IRb
23	138,862 (IGS *trnA-AAC-chIL*)	162,363 (IGS *ndhF -trnA-AAC*)	30	F	IRb; SSC
24	162,363 (IGS *ndhF -trnA-AAC*)	162,363 (IGS *ndhF -trnA-AAC*)	30	P	SSC; SSC
C. taitungensis					
1	90,037 (IGS *trnL-trnH*)	147,394 (*rrn16*)	15830	P	LSC; IRb
2	105,868 (*rrn16*)	138,150 (IGS *chIL-hyperthetical gene*)	9243	P	IRa; IRb
3	28,272 (IGS *rpoB -trnC*)	28,346 (IGS *rpoB -trnC*)	55	F	LSC; LSC
4	33,996 (*hyperthetical gene*)	33,996 (*hyperthetical gene*)	37	R	LSC; LSC
5	74,199 (*clpP*)	74,199 (*clpP*)	33	R	LSC; LSC
6	8,161 (IGS *trnQ-psbK*)	8,161(IGS *trnQ-psbK*)	32	R	LSC; LSC
7	8,161 (IGS *trnQ-psbK*)	8,161 (IGS *trnQ-psbK*)	31	R	LSC; LSC
8	8,161 (IGS *trnQ-psbK*)	8,162 (IGS *trnQ-psbK*)	31	F	LSC; LSC
9	8,162 (IGS *trnQ-psbK*)	8,162 (IGS *trnQ-psbK*)	31	R	LSC; LSC

10	49,666 (*hyperthetical gene*)	49,692 (*hyperthetical gene*)	31	F	LSC; LSC
11	8,161 (IGS *trnQ-psbK*)	8,161 (IGS *trnQ-psbK*)	30	R	LSC; LSC
12	8,161 (IGS *trnQ-psbK*)	8,163 (IGS *trnQ-psbK*)	30	F	LSC; LSC
13	8,163 (IGS *trnQ-psbK*)	8,163 (IGS *trnQ-psbK*)	30	R	LSC; LSC
14	114,863 (IGS *hyperthetical gene-ndhF*)	114,863 (IGS *hyperthetical gene-ndhF*)	30	P	IRa; IRa
15	114,863 (IGS *hyperthetical gene-ndhF*)	138,368 (IGS *hyperthetical gene-chIL*)	30	F	IRa; IRb
16	138,368 (IGS *hyperthetical gene-chIL*)	138,368 (IGS *hyperthetical gene-chIL*)	30	P	IRb; IRb

Table S2. Simple sequence repeats in the *Cycas* chloroplast genomes.

No.	SSR type	SSR	size	start	SSR-containing region
***C. debaoensis* (KM459003)**					
2	p1	A	20	8307	IGS (*trnQ-UUG-psbK*); LSC
	p1	A	20	73072	*clpP*; LSC
1	p1	A	19	123910	IGS (*ndhG-ndhI*); SSC
1	p1	A	14	32963	IGS (*trnE-UUC-trnH-GUG*); LSC
1	p1	A	13	68693	IGS (*tufA-trnH-GUG*); LSC
2	p1	A	11	101909	IGS (*trnH-GUG-rps7*); IRa
	p1	A	11	148076	IGS (*trnV-GAC-rps7*); IRb
2	p1	A	10	1872	*trnK-UUU*; LSC
	p1	A	10	11723	IGS (*trnG-UCC-trnG-UCC*); LSC
1	p1	T	19	15487	IGS (*atpH-atpI*); LSC
1	p1	T	18	10133	IGS (*trnS-GCU-ycf12*); LSC
2	p1	T	16	57647	IGS (*atpB-rbcL*); LSC
	p1	T	16	84487	*rpl16*; LSC
3	p1	T	15	54642	IGS (*trnM-CAU-ndhC*); LSC
	p1	T	15	84793	*rpl16*; LSC
	p1	T	15	134343	IGS (*ycf1-chlN*); SSC
1	p1	T	13	74062	*clpP*; LSC
2	p1	T	12	5951	*rps16*; LSC
	p1	T	12	118367	IGS (*rpl32-trnP-GGG*); SSC
4	p1	T	11	83225	*rps8*; LSC
	p1	T	11	88616	IGS (*rpl23-trnI-CAU*); LSC
	p1	T	11	102857	IGS (*rps7-trnV-GAC*); IRa
	p1	T	11	149024	IGS (*trnV-GAC-rps7*); IRb
3	p1	T	10	63349	IGS (*ycf4-cemA*)LSC
	p1	T	10	69918	IGS (*trnP-UGG-psaJ*); LSC
	p1	T	10	125903	*ndhA*; SSC
1	p1	G	14	70450	IGS (*psaJ-trnH-GUG*); LSC
1	p1	G	11	52763	IGS (*ndhC-trnH-GUG*); LSC
2	p1	G	10	70920	IGS (*rpl33-trnH-GUG*); LSC
	p1	G	10	149993	IGS (*rps7-ndhB*); LSC
1	p1	C	14	44747	*ycf3*; LSC
1	p1	C	11	5229	*rps16*; LSC
2	p1	C	10	17681	IGS (*rps2-rpoC2*); LSC
	p1	C	10	100941	IGS (*ndhB-rps7*); LSC
3	p2	(TA)14	28	29832	IGS (*petN-psbM*); LSC
	p2	(TA)9	18	1453	IGS (*psbA-trnK-UUU*); LSC
	p2	(TA)6	12	15670	IGS (*atpH-atpI*); LSC
1	p2	(GA)6	12	68376	IGS (*tufA-trnH-GUG*); LSC
1	p2	(AT)6	12	15808	IGS (*atpH-atpI*); LSC

No.	SSR type	SSR	size	start	SSR-containing region
***C. revoluta* (NC_020319.1)**					
1	p1	A	18	98528	*orf64a*; LSC
1	p1	A	17	29429	*rpl16*; LSC
3	p1	A	16	29121	*rpl16*; LSC
	p1	A	16	43344	IGS (*rpl33-psaJ*); LSC
	p1	A	16	103892	IGS (*ycf12-tRNA-AGC*); LSC
1	p1	A	15	44058	IGS (*tRNA-CCA-psaJ*); LSC
1	p1	A	14	39873	*clpP*; LSC
3	p1	A	12	59343	*trnA-GUA*; LSC
	p1	A	12	141756	*chIN*; SSC
	p1	A	12	157688	IGS (*trnA-CCC-rps32*); SSC
6	p1	A	11	11048	IGS (*orf30-orf64e*); IRa
	p1	A	11	25180	IGS (*tRNA-AUA-rpl23*); LSC
	p1	A	11	56339	IGS (*rbcL-atpB*); LSC
	p1	A	11	99805	*atpF*; LSC
	p1	A	11	108061	*rps16*; LSC
	p1	A	11	127103	IGS (*rps7-rps12*); IRb
1	p1	A	10	50641	IGS (*cemA-orf73*); LSC
1	p1	T	21	105717	IGS (*psbK-tRNA-CAA*); LSC
1	p1	T	18	40861	*clpP*; LSC
2	p1	T	17	81041	IGS (*tRNA-GAA-psbD*); LSC
	p1	T	17	152152	IGS (*ndhI-ndhG*); SSC
1	p1	T	14	45287	IGS (*petL-psbE*); LSC
1	p1	T	13	43034	IGS (*rps18-rpl33*); LSC
2	p1	T	12	30111	IGS (*rpl16-rpl14*); LSC
	p1	T	12	102305	IGS (*tRNA-AGA-tRNA-GGA*); LSC
4	p1	T	11	45411	IGS (*petL-psbE*); LSC
	p1	T	11	81462	IGS (*tRNA-GAA-psbD*); LSC
	p1	T	11	128053	IGS (*orf64e-orf130*); IRb
	p1	T	11	11998	*orf64d*; IRa
2	p1	T	10	112149	IGS (*matK-tRNA-AAA*); LSC
	p1	T	10	154151	IGS (*psaC-ndhD*); SSC
2	p1	C	10	35519	*petB*; LSC
	p1	C	10	43507	IGS (*rpl33-psaJ*); LSC
1	p2	(AT)16	32	84160	IGS (*psbM-petN*); LSC
1	p2	(AT)6	12	98212	IGS (*atpl-orf64a*); LSC
1	p2	(TA13	26	112562	IGS (*tRNA-AAA-psbA*); LSC
2	p2	(TA)6	12	98350	IGS (*atpl-orf64a*); LSC
	p2	(TC)6	12	45610	IGS (*petL-psbE*); LSC

C. taitungensis (NC_009618)

No.	SSR type	SSR	size	start	SSR-containing region
1	p1	A	32	8162	IGS (*trnQ-psbK*); LSC
2	p1	A	27	34002	*hypothetical gene*; LSC
	p1	A	27	74203	*clpP*; LSC
1	p1	A	21	125086	IGS (*ndhJ-ndhI*); SSC
1	p1	A	16	149205	IGS (*hypothetical gene-hypothetical gene*); IRb
1	p1	A	15	11592	IGS (*trnG-trnR*); LSC
2	p1	A	14	1730	*trnK*; LSC
	p1	A	14	33584	*hypothetical gene*; LSC
3	p1	A	11	72043	IGS (*rps33-rps18*); LSC
	p1	A	11	84973	IGS (*rps14-rps16*); LSC
	p1	A	11	103094	*hypothetical gene*; IRa
1	p1	T	21	15364	*hypothetical gene*; LSC
1	p1	T	20	10000	IGS (*trnS-ycf12*); LSC
2	p1	T	19	85646	*rpl16*; LSC
	p1	T	19	85955	*rpl16*; LSC
1	p1	T	18	71732	IGS (*psaJ-rpl33*); LSC
3	p1	T	16	71020	*hypothetical gene*; LSC
	p1	T	16	104042	IGS (*hypothetical gene-hypothetical gene*); IRa
	p1	T	16	135490	IGS (*ycf1-chlN*); SSC
3	p1	T	14	5828	*rpl16*; LSC
	p1	T	14	75200	*clpP*; LSC
	p1	T	14	119558	IGS (*rpl32-trnP*); SSC
3	p1	T	12	14093	*atpF*; LSC
	p1	T	12	58704	IGS (*atpB-rbcL*); LSC
	p1	T	12	89913	IGS (*rpl23-trnl*); LSC
4	p1	T	11	55702	*trnV*; LSC
	p1	T	11	64402	IGS (*hypothetical gene-cemA*); LSC
	p1	T	11	84373	*rps8*; LSC
	p1	T	11	150158	*hypothetical gene*; IRb
2	p1	T	10	86449	IGS (*rpl16-rps3*); LSC
	p1	T	10	89790	IGS (*rpl23-trnl*); LSC
1	p1	C	14	17560	IGS (*rps2-rpoC2*); LSC
1	p1	C	12	5105	*rps16*; LSC
1	p1	C	10	45810	*ycf3*; LSC
1	p1	G	11	71575	IGS (*psaJ-rpl33*); LSC
1	p2	(TA)13	26	29723	IGS (*petN-hypothetical gene*); LSC
1	p2	(TA)6	12	15549	IGS (*hypothetical gene-atpl*); LSC
1	p2	(AT)6	12	15687	IGS (*hypothetical gene-atpl*); LSC
1	p2	(GA)6	12	69429	IGS (*tufA-hypothetical gene*); LSC

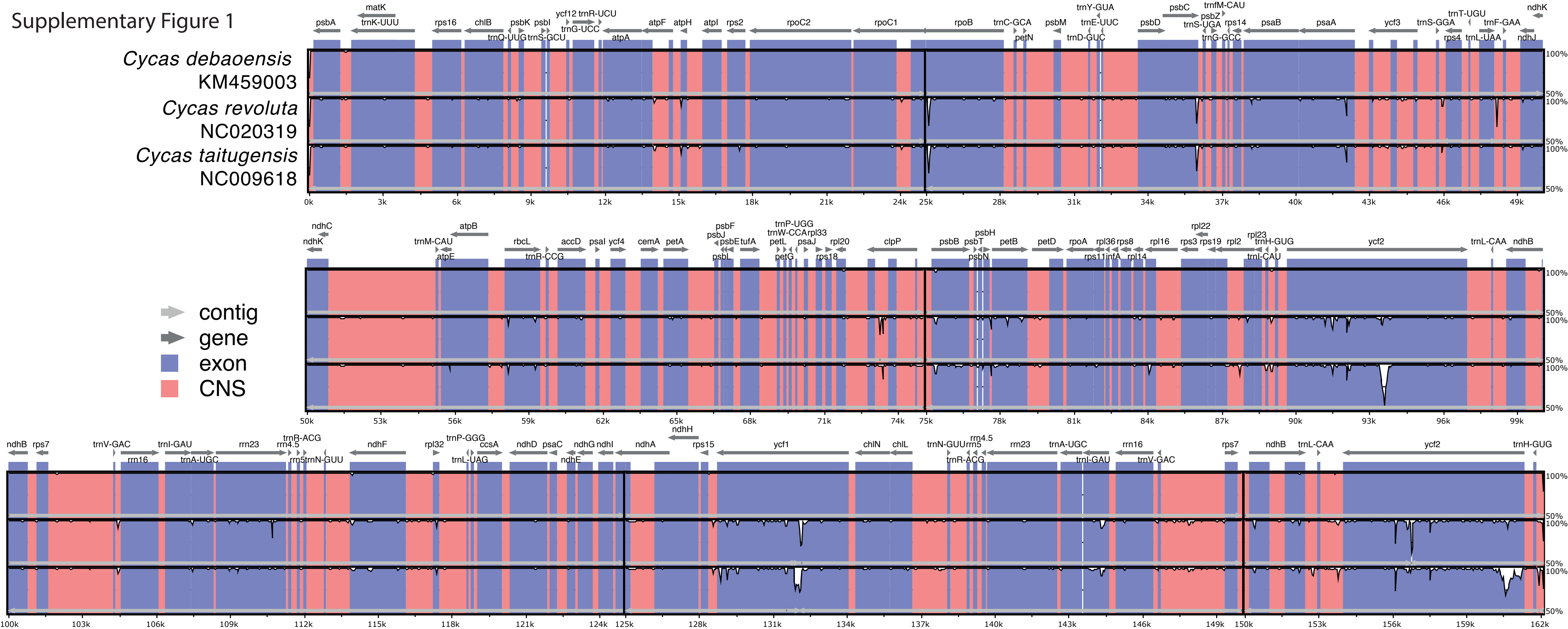

Supplementary Figure 1
